# Supplementary material for: New Self-Report Measures of Commuting Behaviors to University and Their Association with Sociodemographic Characteristics
Source: Int J Environ Res Public Health. 2021 Nov 29;18(23):12557. doi: 10.3390/ijerph182312557 (PMC8657066; doi:10.3390/ijerph182312557)
Supplement: Supplementary file 1 [file ijerph-18-12557-s001.zip › ijerph-1472886-supplementary.pdf]

Supplementary Material

Table S1. Example of commuting energy expenditure per minute and total calculated, based in the Compendium of Physical Activities code for adults, according to the mode of commuting to university.

	Mode of commuting to university		
	**Active**	**Public**	**Private**
**Code of the Compendium**			
EE p/m	2.0 METs (less than 3.0 km/hr)<br>2.8 METs (3.0 to 3.9 km/hr)<br>3.0 METs (4.0 to 4.49 km/hr)<br>3.65 METs (4.5 to 5.49 km/hr)<br>4.3 METs (5.5 to 6.49 km/hr)<br>5 METs (6.5 to 6.9 km/hr<br>7 METs (7 to 8.49 km/hr)<br>8.3 METs (≥ 8.5 km/hr)	(1.3 METs × time to commuting) + (7.5⁎ × 2.5 METs⁑) / time to commuting	1.3 METs
**Calculated**			
Total EE	(METs according to EE p/m × time to commuting)	(1.3 METs × time to commuting) + (7.5⁎ × 2.5 METs⁑)	(1.3 METs × time to commuting)

Notes: EE = Energy expenditure; p/m = per min; PA = physical activity km/hr = kilometers/hours; ⁎ = minutes added per trip; ⁑ = code 17161 walking to the stations and stops.
